# Prognostic Influence of Pre-Operative C-Reactive Protein in Node-Negative Breast Cancer Patients

**DOI:** 10.1371/journal.pone.0111306

**Published:** 2014-10-23

**Authors:** Isabel Sicking, Karolina Edlund, Eva Wesbuer, Veronika Weyer, Marco J. Battista, Antje Lebrecht, Christine Solbach, Marianna Grinberg, Johannes Lotz, Gerald Hoffmann, Jörg Rahnenführer, Jan G. Hengstler, Marcus Schmidt

**Affiliations:** 1 Department of Obstetrics and Gynecology, Johannes Gutenberg University, Mainz, Germany; 2 Leibniz Research Centre for Working Environment and Human Factors (IfADo), Dortmund University of Technology, Dortmund, Germany; 3 Institute of Medical Biometry, Epidemiology and Informatics (IMBEI), Johannes Gutenberg University, Mainz, Germany; 4 Department of Statistics, Dortmund University of Technology, Dortmund, Germany; 5 Institute for Clinical Chemistry, Johannes Gutenberg University, Mainz, Germany; University of Campinas, Brazil

## Abstract

The importance of inflammation is increasingly noticed in cancer. The aim of this study was to analyze the prognostic influence of pre-operative serum C-reactive protein (CRP) in a cohort of 148 lymph node-negative breast cancer patients. The prognostic significance of CRP level for disease-free survival (DFS), metastasis-free survival (MFS) and overall survival (OS) was evaluated using univariate and multivariate Cox regression, also including information on age at diagnosis, tumor size, tumor grade, estrogen receptor (ER), progesterone receptor (PR) and human epidermal growth factor receptor 2 (HER2) status, proliferation index (Ki67) and molecular subtype, as well as an assessment of the presence of necrosis and inflammation in the tumor tissue. Univariate analysis showed that CRP, as a continuous variable, was significantly associated with DFS (P = 0.002, hazard ratio [HR]  = 1.04, 95% confidence interval [CI]  = 1.02–1.07) and OS (P = 0.036, HR  = 1.03, 95% CI  = 1.00–1.06), whereas a trend was observed for MFS (P = 0.111). In the multivariate analysis, CRP retained its significance for DFS (P = 0.033, HR  = 1.01, 95% CI  = 1.00–1.07) as well as OS (P = 0.023, HR  = 1.03, 95% CI  = 1.00–1.06), independent of established prognostic factors. Furthermore, large-scale gene expression analysis by Affymetrix HG-U133A arrays was performed for 72 (48.6%) patients. The correlations between serum CRP and gene expression levels in the corresponding carcinoma of the breast were assessed using Spearman's rank correlation, controlled for false-discovery rate. No significant correlation was observed between CRP level and gene expression indicative of an ongoing local inflammatory process. In summary, pre-operatively elevated CRP levels at the time of diagnosis were associated with shorter DFS and OS independent of established prognostic factors in node-negative breast cancer, supporting a possible link between inflammation and prognosis in breast cancer.

## Introduction

The microenvironment of solid tumors is often rich in inflammatory cells which have appeared as essential players in the tumorigenic process [1]. A protective role of the immune system, especially in early stages of tumorigenesis, has become evident and a link between immune cell-infiltration and better prognosis has been described in various cancer types [2]–[6]. On the other hand, the immune system is known to be able to promote cancer initiation and progression and the causal relationship between chronic inflammation within the local tissue environment and cancer has received increased attention in recent years, leading up to the concept of cancer-related inflammation as an emerging hallmark of cancer [7].

Accordingly, a systemic inflammatory response as shown by an elevated concentration of circulating C-reactive protein (CRP) in peripheral blood, has frequently been associated with increased incidence as well as worse outcome in numerous types of cancer, e.g. gastro-oesophageal cancer, non-small cell lung cancer and prostate cancer [8]–[11]. CRP is a non-specific acute-phase protein that rises on acute infection as well as tissue trauma, chronic inflammatory disease, myocardial infarction, surgery and cancer. It is secreted primarily by hepatocytes in response to cytokine stimulation by for instance IL-1, IL-6 and TNF-alpha [12]. A recent meta-analysis underscored that CRP, as a biomarker of inflammation, is related to impaired outcome also in breast cancer patients [13]. However, this association was not confirmed by others [14], [15]. Only few studies have examined the impact of pre-operative CRP levels on breast cancer prognosis, thus far with mixed results [16]–[20].

The association between CRP level and breast cancer survival has until now been examined mainly in patients with adjuvant systemic treatment. Therefore, the aim of the present study was to analyze the influence of pre-operative CRP level in an untreated cohort of lymph node-negative breast cancer patients in relation to survival and established prognostic factors. Furthermore, we wanted to elucidate potential associations between pre-operative serum CRP and genes expressed in corresponding breast cancer specimen.

## Materials and Methods

### Patient cohort

There were three main eligibility criteria (i) node-negative breast cancer (ii) no systemic treatment in the adjuvant setting (iii) availability of CRP measurement. The initial study cohort consisted of 420 node-negative breast cancer patients, treated at the Department of Obstetrics and Gynecology at Johannes Gutenberg University Mainz between the years 1985 and 2004. Of these 420 patients, pre-operative CRP status was available for 148 individuals treated by surgical tumor resection, either modified radical mastectomy (n = 43; 29.1%) or breast conserving surgery followed by irradiation (n = 105; 70.9%), who did not receive systemic therapy in the adjuvant setting. The median age at diagnosis of the patients was 62 years (range 40 to 90 years). The mean follow-up time was 113 months. All patients provided their informed consent before study inclusion.

Information on tumor size (pT stage) as well as presence of necrosis or inflammation in the tumor was collected from the corresponding pathology report of the Gynecological Pathology Division”. From the breast cancer database [21], information on about of age at diagnosis, histological tumor grade, which was assigned according to Elston and Ellis [22]., estrogen receptor (ER), progesterone receptor (PR) status and human epidermal growth factor receptor 2 (HER2) status and proliferation index (Ki67) were obtained (Table 1). Immunohistochemical analyses for ER, PR, HER2 and Ki-67Ki67 and HER2 were performed on 4-µm-thick sections according to standard procedures. Briefly, serial sections of formalin-fixed and paraffin-embedded tumor tissues were stained with a monoclonal ER antibody (clone 1D5, Dako, Glostrup, Denmark), a monoclonal PR antibody (clone PgR 636, Dako), a monoclonal Ki-67Ki67 antibody (clone MIB-1, Dako, Glostrup, Denmark) as well as a polyclonal HER2 antibody (A0485, Dako, Glostrup, Denmark). HER2 was scored from 0 to 3+ according to the well-published manufacturer's instructions. HER2 3+ tumors were considered HER2 positive. All HER2 2+ cases were confirmed by Fluorescence in-situ hybridization (FISH) using a dual-color probe (DakoCytomation) containing a spectrum orange-labeled HER-2HER2 gene (17q11.2–q12) probe and a spectrum green-labeled centromere control for chromosome 17 (17p11.1–q11.1). HER2 2+ tumors with HER2 amplification were finally considered HER2 positive. ER and PR was analyzed as percentage of all tumor cells and any nuclear expression >0 was considered positive. Ki67 expression in more than 20% of nuclei was considered as high expression (highly proliferative) and a percentage ≤20% was defined as low expression. Building on these variables, we calculated molecular subtypes: Luminal A (ER+ and/or PR+, HER2−, Ki67≤20%), Luminal B (ER+ and/or PR+, HER2−, Ki67>20%), Basal-like (ER− and PR−, HER2−), and HER2+ (ER+/−, PR+/−, HER2+).

**Table 1:** Clinicopathological characteristics for node-negative breast cancer patients.

	n	%
**Total number of patients**	148	100.0
**C-reactive protein (CRP)**		
Elevated (>5mg/l)	31	20.9
Normal (≤5mg/l)	117	79.1
**Tumor size**		
≤2cm	105	70.9
>2cm	43	29.1
**Tumor grade**		
Grade I	40	27.0
Grade II	84	56.8
Grade III	24	16.2
**Age at diagnosis**		
<50 years	29	19.6
≥50 years	119	80.4
**Estrogen receptor status**		
ER positive	128	86.5
ER negative	17	11.5
Missing data	3	2.0
**Progesterone receptor status**		
PR positive	33	22.3
PR negative	112	75.7
Missing data	3	2.0
**HER2 status**		
HER2 positive	16	10.8
HER2 negative	129	87.2
Missing data	3	2.0
**Ki67**		
≤20%	94	63.5
>20%	43	29.1
Missing data	11	7.4
**Molecular subtype**		
Luminal A	86	58.1
Luminal B	24	16.2
Basal-like	13	8.8
HER2+	16	10.8
Missing data	9	6.1
**Local recurrence**		
Yes	10	6.8
No	138	93.2
**Metastasis**		
Yes	20	13.5
No	128	86.5
**Cause of death**		
Breast cancer	13	8.8
Other	21	14.2
Alive at last follow-up	114	77.0
**Necrosis**		
Absent	110	74.3
Present	48	25.7
**Inflammation**		
Absent	98	66.2
Present	50	33.8

Gene expression array data from matched tumor tissue, analyzed by Affymetrix GeneChip Human Genome U133A arrays, was available for a subset of patients (n = 72) as previously described [2]. Raw.cel files, MAS 5.0 processed data and patient data have been deposited in National Center for Biotechnology Information (NCBI) Gene Expression Omnibus (GEO) and are accessible through GEO Series accession no. GSE11121. We documented death from cancer or from other reasons unrelated to breast cancer and recurrence of disease, which include metastasis and local relapse. 13 (8.8%) patients died from breast cancer, 21 (14.2%) patients died from causes unrelated to breast cancer, 114 (77.0%) patients were alive at the date of last follow-up, 10 (6.8%) patients suffered from locally-recurrent disease and 20 (13.5%) developed distant metastasis. Patients who died from other reasons were censored from the survival analysis at the date of death. All participants provided written informed consent which was documented in the patient file. This study as well as the consent procedure was approved by the ethical review board of the medical association of Rhineland-Palatinate. All clinical investigations were conducted according to the principles expressed in the Declaration of Helsinki. The manuscript was prepared in agreement with the reporting recommendations for tumor marker reporting studies [23]. Ethical standards: The experiments comply with the current laws of Germany.

### C-reactive protein

A 10-mL blood sample was collected the day before surgery. The sample was processed within 3 hours of collection. During the period of observation, the method of CRP determination changed once in 1992 from a nephelometric to a turbidimetric assay (Behring Diagnostics, Marburg, Germany and Boehringer Mannheim/Roche Diagnostics, Mannheim, Germany, respectively). The assays were performed following the manufacturer's instructions on the BN-systems (Behring Diagnostics, Marburg, Germany) and the Hitachi 717/747/917 (Boehringer Mannheim/Roche Diagnostics, Mannheim, Germany) automated analyzers, respectively. According to the methodological progress, a wide range CRP assays was introduced in the late 90s/early 2000s. Since then, a measuring range of 0.1–300 mg/l was used. During the whole period, the upper limit of normal was 5 mg/l for all assays used. Intra- and inter-assay imprecision was less than 5% for CRP. Control materials were used for running each assay for quality control purposes.

### Statistical analysis

To evaluate the association between pre-operative CRP level and established prognostic factors such as age, tumor size, tumor grade, ER, PR and HER2 status, proliferation index (Ki67) and molecular subtype with survival time, uni- and multivariate Cox regression analyses were performed. The internal stability of the models was tested by bootstrap resampling [24]–[27]. Briefly, new data sets with the same size of the original one were created by random sampling with replacement. We calculated 10.000 Bootstrap samples from the original data set followed by the same Cox regression. To avoid potential pitfalls of dichotomization of prognostic factors with concomitant loss of test power [28]–[30] we used the prognostic factors in our multivariate Cox regression model as continuous variables. Survival rates were calculated according to the Kaplan-Meier method and survival times were compared with the Log-rank test. Disease-free survival (DFS) was specified the time between the date of surgery and the date of loco-regional or metastatic recurrence, breast cancer-related death or last follow-up. Metastasis-free survival (MFS) was defined as the time between date of surgery and diagnosis of distant metastasis. Overall survival (OS) was defined as the time between the date of surgery and the date of death. The Wilcoxon rank-sum test was applied to identify significant correlations between CRP level and clinical variables. A potential correlation between pre-operative CRP level and expression of metagenes representative of T-and B-cell tumor infiltration, tumor cell proliferation and estrogen receptor positivity [2] was assessed using Spearman's rank correlation coefficient. Additionally, potential correlations between pre-operative CRP level and mRNA expression level for all probe sets on the Affymetrix HG-U133A array were assessed, with the false discovery rate adjusted to 0.05 according to Benjamini and Yekutieli. All P values were two sided. Since, except for the correlations between CRP and Affymetrix probe sets, no correction for multiple testing was performed, all results were interpreted as explorative. Statistical analyses were performed using the freely available statistical computing language R3.0.1.

## Results

The pre-operatively assessed CRP level in peripheral blood was in the majority of patients (79.1%) within the normal reference interval, conventionally defined as ≤5 mg/l, while a subset of patients (20.9%) displayed an elevated CRP level (>5 mg/l) (Table 1).

**Figure 1:**
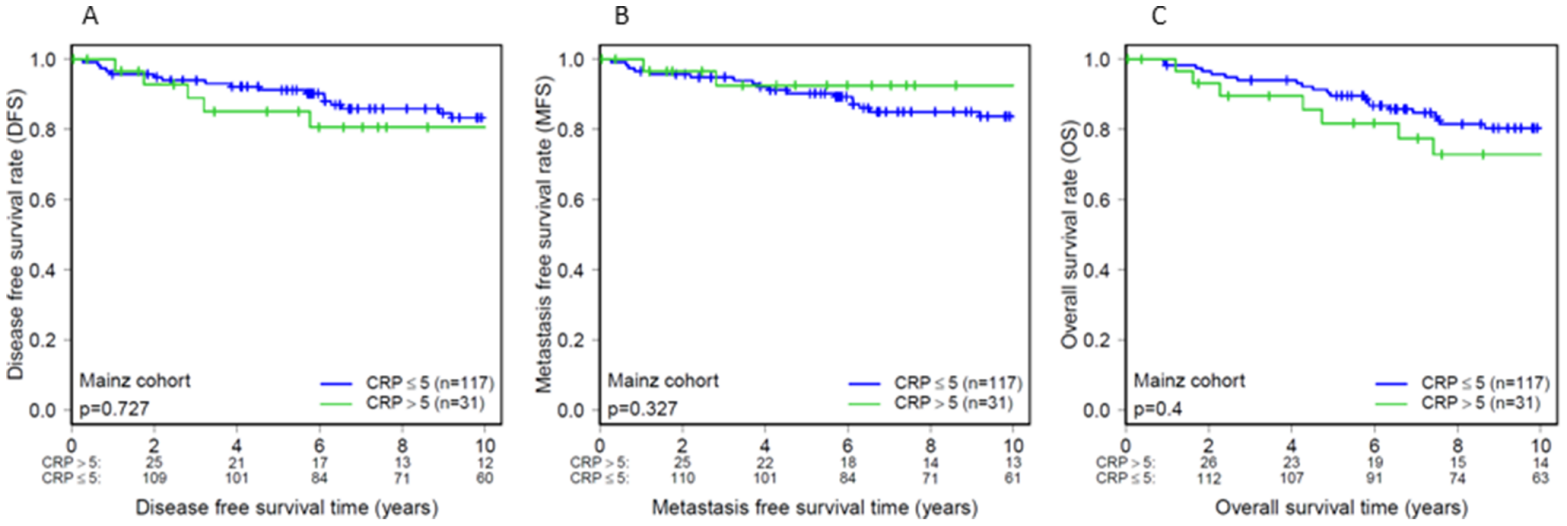
Association of CRP with survival in node-negative breast cancer (n = 148). CRP dichotomized using values above upper limit normal (>5 mg/l; n = 31) shows no significant association with disease-free survival (A), metastasis-free survival (B) and overall survival (C), respectively. The numbers below the diagrams represent patients at risk at the time point indicated on the x-axis.

Initially, univariate Cox regression analysis was performed to assess the impact on survival time of pre-operative CRP level, age at diagnosis, tumor size, tumor grade, ER, PR and HER2 status, proliferation index (Ki67), molecular subtype and presence of necrosis and inflammation in the tumor tissue (Table 2). In univariate analysis, elevated pre-operative CRP levels were associated with shorter DFS (P = 0.002, HR  = 1.04, 95% CI  = 1.02–1.07) and OS (P = 0.036, HR  = 1.03, 95% CI  = 1.00–1.06), while a non-significant trend was observed for MFS (P = 0.111, HR  = 1.03 95% CI  = 0.99–1.07) (Table 2). The relatively small hazard ratios are explained by the fact that CRP was statistically analyzed as a continuous variable with a relatively wide dynamic range (1–67 mg/l). To envision the prognostic impact of pre-operative CRP level, we performed Kaplan-Meier analysis to compare patients with a CRP level within the normal reference interval (≤5 mg/l) with the patient subset displaying elevated CRP (>5 mg/l), however revealing a non-significant association at this cut-off (Figure 1A–C).

**Table 2:** Univariate Cox regression analysis for disease-free survival (DFS), metastasis-free survival (MFS) and overall survival (OS).

		DFS			MFS			OS		
		HR	CI	*p-*value	HR	CI	*p-*value	HR	CI	*p-*value
**CRP**	mg/L	1.04	1.02–1.07	0.002	1.03	0.99–1.07	0.110	1.03	1.00–1.06	0.036
**Age at diagnosis**	≥50 years	ref								
	<50 years	0.41	0.18–0.93	0.032	0.29	0.12–0.71	0.006	1.29	0.53–3.12	0.575
**Tumor size**	≤2cm	ref								
	>2cm	2.23	1.00–4.98	0.050	1.76	0.72–4.31	0.216	2.03	1.03–3.99	0.041
**Tumor grade**	I–II	ref								
	III	3.78	1.65–8.65	0.002	4.00	1.63–9.80	0.002	3.12	1.51–6.46	0.002
**ER status**	Negative	ref								
	Positive	0.42	0.16–1.12	0.083	1.05	0.24–4.52	0.950	0.55	0.23–1.34	0.190
**PR status**	Negative	ref								
	Positive	0.46	0.20–1.05	0.066	0.86	0.31–2.38	0.777	0.56	0.27–1.13	0.106
**HER2 status**	Negative	ref								
	Positive	3.29	1.30–8.31	0.012	4.15	1.59–10.81	0.004	2.22	0.96–5.12	0.061
**Proliferation**	Ki67≤20%	ref								
	Ki67>20%	2.17	0.96–4.93	0.063	1.70	0.68–4.23	0.253	1.58	0.78–3.20	0.203
**Necrosis**	Absent	ref								
	Present	1.40	0.60–3.27	0.440	1.86	0.76–4.56	0.173	1.21	0.59–2.50	0.604
**Inflammation**	Absent	ref								
	Present	1.15	0.50–2.62	0.743	0.82	0.31–2.13	0.682	1.20	0.60–2.39	0.614
**Molecular subtype**	Luminal A	ref								
	Luminal B	0.91	0.26–3.28	0.890	1.14	0.31–4.21	0.843	1.02	0.37–2.77	0.976
	Basal-like	3.16	1.00–9.97	0.049	1.79	0.39–8.30	0.457	2.05	0.68–6.14	0.200
	HER2-like	3.63	1.34–9.85	0.011	4.33	1.54–12.20	0.005	2.50	1.03–6.11	0.044

HR: Hazard Ratio; CI: 95% Confidence Interval; Luminal A: ER+ and/or PR+, HER2−, Ki67≤20%; Luminal B: ER+ and/or PR+, HER2−, Ki67>20%; Basal-like: ER− and PR−, HER2−; HER2: ER+/−, PR+/−, HER2+.

**Figure 2:**
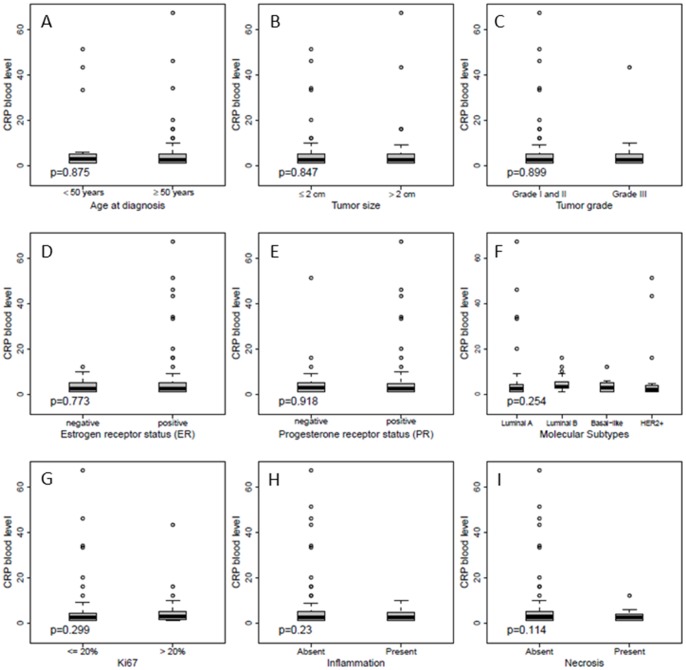
Association of CRP with clinico-pathological factors in node-negative breast cancer (n = 148). Wilcoxon rank-sum test between pre-operative CRP level (mg/l) and age at diagnosis (A), tumor size (B), tumor grade (C), estrogen receptor status (D), progesterone receptor status (E), molecular subtypes (F), proliferation (G), inflammatory infiltrate (H) and necrosis (I) showing no significant associations.

In multivariate Cox analysis, including factors that showed an impact on prognosis in the univariate analysis, an elevated pre-operative CRP level remained significantly associated with shorter DFS (P = 0.033, HR  = 1.03, 95% CI  = 1.00–1.07) and OS (P = 0.023, HR  = 1.03, 95% CI  = 1.00–1.06), independent of established prognostic factors, whereas CRP failed to show an independent association with MFS (P = 0.469, HR  = 1.01, 95% CI  = 0.98–1.05) (Table 3). Similar results were obtained for DFS when the Cox analysis was adjusted to non-dichotomized/continuous variables (Table S1 and S2). The internal stability of the models was tested by bootstrapping (Figure S1). Only the analysis of OS resulted in a 95% interval higher than one [1.0; 1.07] while the histograms for DFS and MFS showed relatively long tails on the left side (Figure S1). Moreover, pre-operative CRP levels did not correlate with age at diagnosis, tumor size, histological tumor grade, ER, PR status or HER2 status, proliferation index (Ki67), molecular subtypes, Ki-67, necrosis or inflammatory infiltrate (Figure 2A–I).

**Table 3:** Multivariate Cox regression analysis for disease-free survival (DFS), metastasis-free survival (MFS) and overall survival (OS).

		DFS			MFS			OS		
		HR	CI	*p-*value	HR	CI	*p-*value	HR	CI	*p-*value
**CRP**	mg/L	1.03	1.00–1.07	0.033	1.01	0.98–1.05	0.469	1.03	1.00–1.06	0.023
**Age at diagnosis**	≥50 years	ref								
	<50 years	0.41	0.17–1.02	0.055	0.29	0.11–0.75	0.011	1.56	0.61–4.00	0.352
**Tumor size**	≤2cm	ref								
	>2cm	2.47	1.06–5.76	0.036	2.11	0.81–5.53	0.128	1.82	0.88–3.79	0.108
**Tumor grade**	I–II	ref								
	III	3.07	0.98–9.57	0.054	2.89	0.83–10.09	0.097	4.67	1.62–13.46	0.004
**Molecular subtype**	Luminal A	ref								
	Luminal B	0.58	0.13–2.64	0.483	0.83	0.16–4.15	0.815	0.42	0.12–1.43	0.164
	Basal-like	2.13	0.52–8.70	0.290	1.24	0.21–7.36	0.813	0.80	0.20–3.11	0.742
	HER2-like	2.12	0.59–7.55	0.247	2.86	0.73–11.26	0.133	1.22	0.41–3.63	0.725

HR: Hazard Ratio; CI: 95% Confidence Interval; Luminal A: ER+ and/or PR+, HER2−, Ki67≤20%; Luminal B: ER+ and/or PR+, HER2−, Ki67>20%; Basal-like: ER− and PR−, HER2−; HER2: ER+/−, PR+/−, HER2+.

We then wanted to elucidate potential relations between pre-operative CRP level measured in peripheral blood, as a marker of systemic inflammation, and ongoing processes in the local tumor tissue environment. Whole-genome gene expression array data can be considered to represent a snapshot of the tumor transcriptome and was available for 72 patients of the 148 patients included in the study. Including 22,283 probe sets in the analysis, a positive correlation between CRP and mRNA expression level and a p-value ≤0.05 was observed for 165 probe sets (150 genes after converting probe sets to gene symbols using the R package hgu133a and removing duplicates); however after adjustment for multiple testing no gene remained significant (Table S3). Neither were any significant correlation revealed to the expression of previously published metagenes that comprise mRNA-based information representative of tumor infiltration of B and T cells, respectively, tumor cell proliferation and estrogen receptor positivity [2] (Table 4).

**Table 4:** Spearman's rank correlation between pre-operative CRP level in peripheral blood and metagene expression in the tumor tissue.

		rho	*p*-value
**All patients**	T-cell metagene	0.063	0.596
	B-cell metagene	−0.061	0.611
	Proliferation metagene	0.026	0.828
	ER metagene	0.009	0.938
**ER+/HER2−**	T-cell metagene	0.140	0.270
	B-cell metagene	−0.012	0.925
	Proliferation metagene	0.162	0.201
	ER metagene	−0.127	0.317

## Discussion

Prognostic factors for breast cancer patients at the time of diagnosis are today primarily based on clinicopathological characteristics, such as tumor size, axillary lymph node status, and histological differentiation grade, together with estrogen receptor (ER) and epidermal growth factor receptor 2 (HER2) status. Improved prognostication is necessary to decide whether adjuvant chemotherapy is required, in particular for patients with positive hormone receptor status and for node-negative patients [31]. While commercially available gene expression-based prognostic assays, such as Mammaprint [32], Oncotype DX [33] and EndoPredict [34] have proven effective, these are not yet established in clinical routine for every patient. Prognostic biomarkers based on easily implementable non-invasive procedures, which are used in clinical routine, would potentially be of benefit to larger patient groups.

The exact mechanism by which elevated CRP is linked to poor prognosis remains elusive. Recent studies have suggested that elevated biomarkers of systemic inflammation may merely reflect the aggressiveness of the tumor and therefore represent a consequence of established prognostic factors, such as tumor size and grade [17]. This finding is compatible with the observation that cancer patients repeatedly show higher CRP levels than healthy controls [35], [36] and that patients with advanced breast cancer show elevated CRP levels as a sign of increased tumor burden [20], [37]. Another hypothesis is that systemic inflammation may not solely be a consequence of tumor burden, but actively contribute to tumor progression. This view is supported by another study, describing the association between elevated CRP up to 31 months after tumor removal and shorter survival time [38].

In this study we demonstrate that marked elevation of pre-operatively measured CRP level in peripheral blood is associated with shorter disease-free and overall survival in a population of untreated node-negative breast cancer, independent of well-established clinicopathological criteria. Our study has several strengths: (i) it is the first analysis of a patient population not treated with systemic adjuvant therapy which allows for assessment of the pure prognostic effect without potential predictive interactions, (ii) this analysis is – to the best of our knowledge – the first to examine the correlation of CRP values with the expression of a multitude of genes in the corresponding carcinomas of the breast. In this context, our findings reinforce the observed lack of strong correlations between CRP level in peripheral blood and well-known prognostic markers of inflammation, immune response and proliferation in the local tumor tissue environment. This finding shows that blood CRP level is an independent marker of prognosis, but also raises important concerns that systemic inflammation primarily is indicative of co-occurring disease unrelated to the primary tumor in this patient population.

Nonetheless, a number of essential limitations to this study must be considered: (i) the result is based on analysis of one relatively small single center study cohort, as no independent untreated node-negative validation cohort was available, (ii) CRP data was collected retrospectively and was measured only once for each patient, therefore being liable to fluctuations, e.g. due to acute infection, and (iii) lifestyle factors that may influence CRP levels, such as smoking habit, body mass index and comorbidities like diabetes or cardiovascular disease, were not taken into account, as this information was not available for the complete patient cohort. The observation that several patients among those with an marked elevation of pre-operative CRP level died because of reasons other than breast cancer may suggest that elevated CRP primarily represents the presence of concurrent disease in a small patient subset and is unrelated to primary tumor burden in node-negative breast cancer patients, further supported by the lack of correlation to tumor size and grade. In addition, no significant association was observed with metastasis-free survival, possibly suggesting that while systemic inflammation is linked to overall survival, as well as disease-free survival, it is not directly influencing tumor progression and metastasis.

In conclusion, we show that systemic inflammation, assessed by pre-operative CRP level at the time of diagnosis, may influence overall and disease-free survival in untreated node-negative patients, independent of tumor size, tumor grade, and molecular subtype. Based on large-scale gene expression analysis in a subgroup of 72 (48.6%) patients, we highlight for the first time, that circulating CRP is not related to the expression of genes in the corresponding carcinomas of the breast. Nevertheless, the proposed prognostic relevance of pre-operative CRP must be interpreted with caution. Further large-scale prospective investigations will be necessary in order to validate the effect of systemic inflammation on breast cancer prognosis as well as the potential clinical implementation of pre-operative CRP level as a prognostic biomarker in node-negative breast cancer.

## Supporting Information

Figure S1 **Test of the internal stability of the models shown in** **Table 3** **using bootstrapping.** DFS: disease free survival; MFS: metastasis free Survival; OS: overall survival; HR: hazard ratio.
(TIF)

Table S1 **Multivariate Cox regression analysis for disease-free survival (DFS), metastasis-free survival (MFS) and overall survival (OS) based on continuous variables for CRP, age, tumor size and tumor grade.**
(DOC)

Table S2 **Univariate Cox regression analysis for disease-free survival (DFS), metastasis-free survival (MFS) and overall survival (OS) for the non-dichotomized data of Ki67.**
(DOC)

Table S3 **Spearman's rank correlation coefficient between pre-operative CRP level and mRNA expression level for all probe sets on the Affymetrix HG-U133A array was assessed with the false discovery rate (0.05) controlled according to Benjamini and Yekutieli.**
(XLSX)
