# Supplementary figures and images for: Prognostic Influence of Pre-Operative C-Reactive Protein in Node-Negative Breast Cancer Patients
Source: PLoS One. 2014 Oct 23;9(10):e111306. doi: 10.1371/journal.pone.0111306 (PMC4207815; doi:10.1371/journal.pone.0111306)

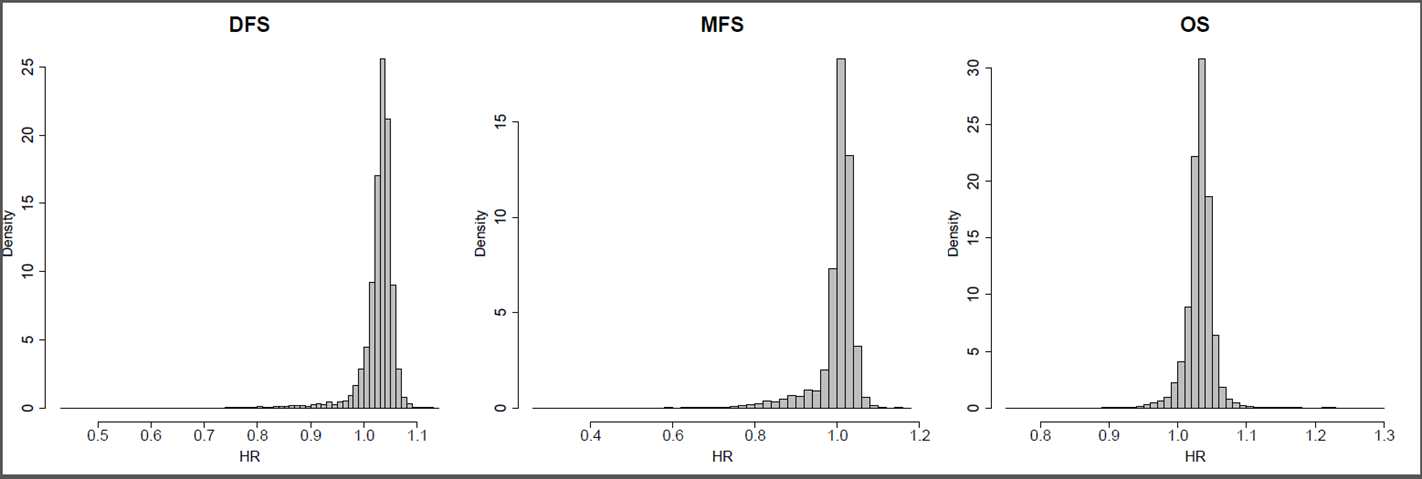

Supplement: Figure S1 — Test of the internal stability of the models shown in Table 3 using bootstrapping. DFS: disease free survival; MFS: metastasis free Survival; OS: overall survival; HR: hazard ratio. (TIF) [file pone.0111306.s001.tif]
