# Supplementary material for: Prognostic Influence of Pre-Operative C-Reactive Protein in Node-Negative Breast Cancer Patients
Source: PLoS One. 2014 Oct 23;9(10):e111306. doi: 10.1371/journal.pone.0111306 (PMC4207815; doi:10.1371/journal.pone.0111306)
Supplement: Table S1 — Multivariate Cox regression analysis for disease-free survival (DFS), metastasis-free survival (MFS) and overall survival (OS) based on continuous variables for CRP, age, tumor size and tumor grade. (DOC) [file pone.0111306.s002.doc]

*Table S1:*

Multivariate Cox regression analysis for disease-free survival (DFS), metastasis-free survival (MFS) and overall survival (OS) based on continuous variables for CRP, age, tumor size and tumor grade

|  |  |  | **DFS** |  |  | **MFS** |  |  | **OS** |  |
| --- | --- | --- | --- | --- | --- | --- | --- | --- | --- | --- |
|  |  | **HR** | **CI** | ***p-*value** | **HR** | **CI** | ***p-*value** | **HR** | **CI** | ***p-*value** |
| **C-reactive protein (CRP)** | mg/L | 1.03 | 1.00-1.06 | 0.028 | 1.02 | 0.98-1.06 | 0.307 | 1.02 | 0.99-1.05 | 0.220 |
| **Age at diagnosis** | years | 0.97 | 0.94-1.01 | 0.178 | 0.96 | 0.92-1.01 | 0.081 | 1.07 | 1.03-1.10 | <0.001 |
| **Tumor size** | T1,2,3 | 2.36 | 1.21-4.59 | 0.011 | 2.08 | 0.99-4.35 | 0.052 | 1.63 | 0.84-3.15 | 0.147 |
| **Tumor grade** | GI, II, III | 2.29 | 0.93-5.61 | 0.070 | 2.62 | 0.98-7.02 | 0.056 | 5.11 | 2.11-12.36 | <0.001 |
| **Molecular subtype** | Luminal A | ref |  |  |  |  |  |  |  |  |
|  | Luminal B | 0.57 | 0.13-2.43 | 0.446 | 0,66 | 0.14-3.02 | 0.588 | 0,30 | 0.09-1.03 | 0.057 |
|  | Basal-like | 1,99 | 0.52-7.66 | 0.319 | 0,95 | 0.17-5.35 | 0.951 | 0,58 | 0.14-2.3 | 0.445 |
|  | HER2 positive | 2,09 | 0.62-7.08 | 0.237 | 2,15 | 0.59-7.83 | 0.245 | 1,25 | 0.40-3.86 | 0.705 |

HR: Hazard Ratio; CI: 95% Confidence Interval; Luminal A: ER+ and/or PR+, HER2-, Ki67≤20%; Luminal B: ER+ and/or PR+, HER2-, Ki67>20%; Basal-like: ER- and PR-, HER2-; HER2: ER+/-, PR+/-, HER2+.
