# Supplementary material for: Prognostic Influence of Pre-Operative C-Reactive Protein in Node-Negative Breast Cancer Patients
Source: PLoS One. 2014 Oct 23;9(10):e111306. doi: 10.1371/journal.pone.0111306 (PMC4207815; doi:10.1371/journal.pone.0111306)
Supplement: Table S2 — Univariate Cox regression analysis for disease-free survival (DFS), metastasis-free survival (MFS) and overall survival (OS) for the non-dichotomized data of Ki67. (DOC) [file pone.0111306.s003.doc]

*Table S2:*

Univariate Cox regression analysis for disease-free survival (DFS), metastasis-free survival (MFS) and overall survival (OS) for the non-dichotomized data of Ki67. The corresponding data for dichotomized Ki67 are shown in Table 2.

|  |  |  | **DFS** |  |  | **MFS** |  |  | **OS** |  |
| --- | --- | --- | --- | --- | --- | --- | --- | --- | --- | --- |
|  |  | **HR** | **CI** | ***p-*value** | **HR** | **CI** | ***p-*value** | **HR** | **CI** | ***p-*value** |
| **Proliferation** | Ki67 % | 1.66 | 1.10-2.51 .2.2...22222222.2.511.10-2.51 | 0.02 | 1.54 | 0.98-2.42 | 0.06 | 1.48 | 1.05-2.08 22222222222222.08222222222222.08 | 0.03 |

HR: Hazard Ratio; CI: 95% Confidence Interval;
